# Supplementary material for: Participation and adherence to mammography screening in the Capital Region of Denmark: The importance of age over time
Source: PLoS One. 2023 Jan 23;18(1):e0280790. doi: 10.1371/journal.pone.0280790 (PMC9870097; doi:10.1371/journal.pone.0280790)
Supplement: Attachment — Submitted filename: Response to the reviewers.docx [file pone.0280790.s001.docx]

We thank the reviewers for their comments to our manuscript and you will find our answers below.

*Have the authors made all data underlying the findings in their manuscript fully available?*

Answer: We have added the following statements to the Method section:

Data from this study are stored in Statistics Denmark, which only can be accessed given the relevant data permits in accordance with GDPR and Danish Law. The data is based on personal identification numbers and open access would seriously compromise the privacy of the women included in the study.

**Reviewer #1:**

*However, I am not clear regarding one of their attempts to explain why the most significant change occurred within the older age groups who participated to a larger extent than they did before, that being since the women aged by approximately 10 years during the study period, the higher participation is coherent with their slightly higher participation rate as younger women.*

Answer: Thank you for that comment and we agree that this is very unclear and we have rewritten the text as follows:

“Women in the youngest age group had over time an increased participation, which possibly influenced their adherence as they aged and moved into older age groups.”

*I don't see from the results the authors' statement in the conclusion that adherence was lower than the recommended 70%.*

Answer: This is indicated in Table 2 in the Results. However to make it clearer we have changed the sentence in Conclusion as follows:

“Complete adherence was lower than the recommended 70%, …”

**Reviewer #2:**

1. *Authors state that this is analysis of registry data, according to me even this requires approval of the ethics committee, as authors state that Danish law does not require the approval, in that case it’s the ethical committee which should issue the waiver.*

Answer: We have been informed that the statement we have provided in the manuscript regarding the need for ethics approval and/or review by a data protection committee satisfies the journal’s policies for research involving human participants.

*2. This is a longitudinal data over 20 year period, which is divided in 5 year age groups, during the 20 year period the participant would have moved from first group to nearly the last group, how is the movement of individual participant from one age group to other over subsequent years handled in the study?*

Answer: Thank you for this comment. This is longitudinal data over a 10 year period and the women will thereby have moved 1 to 2 age groups over the study period. They will have been screened a maximum of 5 times. Our aim was to assess adherence over time by age group which necessitates that women are included each time they are screened and not as a single case. We hope that the reviewer can accept this explanation.

*3. Number of women participated in the program be separated from number of mammograms, as the mammograms will be repeated over period of time while number of eligible women will increase by addition of newer women becoming eligible.*

Answer: Please see the explanation under point 2.

*4. Each women should be treated as a single case even if she underwent screening 10 times, this has not happened in this paper as all women in each round are counted this would mean a person may have been counted 10 times in 20 year period, the data should be separated.*

Answer: Please see the explanation under point 2.

*5. There is no description of eligible population even number of women eligible at a point and overall (eligible in first year +added in second/third etc.)*

Answer: In Table 1 the absolute number of invited women and participating women are given for each age group and each invitation round. In view of our aim to assess adherence, we hope this answer is acceptable to the reviewer.

*6. It is not clear that the participation rates are for all eligible women or all women invited, authors should provide denominator and numerator beside percentage that is provided in text, it is important to mention how many women were eligible at a particular time*

Answer: Please see under point 5.

*7. Has the reason for not participating recorded? Was invitation sent only once or was repeated?*

Answer: We have not recorded the reason for not attending in this study. We have added information regarding reminders as follows:

“Women who do not show up are sent a reminder.”

*8. Were any breast cancer awareness program or educational programs conducted between the study period? Were there any efforts to disseminate the importance of breast cancer screening and mammography? if so, could the increase be because of increase awareness*.

In Denmark all women aged 50-69 are individually invited biennially to screening with mammography. At that time they receive information about advantages and risks with screening in order for them to make an informed choice of whether to participate or not. No regional or national campaigns have existed in the study period.

*9. Did this increased participation resulted in increased detection (change in incidence)? Or stage shift?*

Answer: To assess the detection rate is beyond the scope of this study, as our aim was to assess adherence to the program over time by age group.

*10. Did this increased participation also resulted in increased rate of biopsies?*

Answer: Please see the answer above.

*11. Most importantly did the mortality from breast cancer reduced as a result of increased participation in mammography screening?*

Answer: Certainly the aim with screening is to lower the mortality from breast cancer in the population, but mortality rate is a long time measure, so in the meantime we use short time indicators, out of which one is to study participation and adherence, see European guidelines for quality assurance in breast cancer screening and diagnosis.

*12. What was the benefit of the program and increased participation rate that this article shows?*

Answer: The study points to the change of age as being a stratifying factor for participation to a more equal participation in all age groups, and thereby suggests that other factors might be more determining, and more personalized screening might be beneficial.

*13. Though this study make us understand the age as important factor for participation in program, it does not inform what is the importance of this observation and also the remedies that may improve participation of younger women in breast cancer screening program (or no importance)*

Answer: In Denmark there is no recommendation that women younger than 50 should be offered screening. Within the youngest age group are the first time invitees and therefore there are a certain group that will opt out of the program and will not be invited further. This is explained in the first paragraph of the Discussion.

*14. It is suggested that authors look at additional data and come out with newer findings (or benefits as sought above) that can help in improving the program and reducing the mortality from breast cancer, in its present form, the information provided in the manuscript is already known.*

Answer: We thank the reviewer for this comment and more research is certainly ongoing.
